# Country of Origin of Medical Products and Risk of Labour Rights Abuse: A Cross-Sectional Analysis Using Four Procurement Datasets

**DOI:** 10.7759/cureus.54258

**Published:** 2024-02-15

**Authors:** Jasmin Abbott, Chantelle Rizan, James N Smith, Merete Loeken, Mei L Trueba, Mahmood F Bhutta

**Affiliations:** 1 Internal Medicine, University Hospitals Sussex National Health Service (NHS) Foundation Trust, Brighton, GBR; 2 Global Health and Infection, Brighton and Sussex Medical School (BSMS), Brighton, GBR; 3 Public Health and Primary Care, University of Cambridge, Cambridge, GBR; 4 Category Analysis, The Norwegian Hospital Procurement Trust, South-Eastern Norway Division, Vadsø, NOR; 5 Otolaryngology - Head and Neck Surgery, University Hospitals Sussex National Health Service (NHS) Foundation Trust, Brighton, GBR

**Keywords:** sustainable healthcare system, healthcare policy and management, occupational health and safety, transparency, risk assessment tools, health system research, supply chain management, ethical procurement, healthcare supply chains

## Abstract

Background

Case studies have highlighted labour rights abuse in the manufacture of several healthcare products, but little is known about the scale of the problem or the specific products involved. We aimed to quantify and compare the overall and product-specific risks of labour rights abuse in the manufacture of healthcare products supplied to high-income settings using multiple datasets on the product country of origin (COO).

Methods

Public procurement data from South-Eastern Norway (n=23,972 products) were compared to datasets from three other high-income settings: procurement data from Cambridge University Hospitals, trade data from UN Comtrade, and registry data from the US Food and Drug Administration (FDA). In each dataset, the product COO was matched to the International Trade Union Confederation risk rating for labour abuse and deemed high-risk when rated 4, 5, or 5+.

Results

In the Norway data, 55.4% of products by value had a COO declared, 49.1% of which mapped as high-risk of labour rights abuses. COO was identified for 70/100 products in the Cambridge data, with COO matching high-risk at 59.9% by value. The level of risk for specific medical product categories varied between the Norway, US FDA, and UN Comtrade datasets, but those with higher proportional risk included medical/surgical gloves and electrosurgical products.

Conclusion

Evidence of high-risk of labour rights abuse in the manufacture of healthcare products present in these data indicates a likely high level of risk across the sector. There is an urgent need for global legislative and political reform, with a particular focus on supply chain transparency as a key mechanism for tackling this issue.

## Introduction

Reports in the last 15 years have documented multiple cases of labour rights abuses in the manufacture of medical products. This includes sweatshop and child labour in the manufacture of steel surgical instruments in Pakistan [1] and endemic forced migrant labour in medical glove manufacturing in Malaysia [2]. It also includes state-sponsored forced labour in China: in the manufacture of masks in the Xinjiang province using forced Uyghur labour and of medical gowns in Dandong using alleged forced North Korean labour [3]. Together, these reports suggest labour abuse could be widespread in the medical product sector. However, risk analysis of labour rights abuses in this sector to date has been limited [4], despite medical goods being amongst the top 20 traded globally, with annual trade in 2021 estimated at US$150 billion [5].

Medical products have unique specifications, and supply chains have a mix of public and private stakeholders, yet the impact of these factors on the risk of labour rights abuse is poorly understood. Assumptions of risk made in other sectors [6] should not be presumed. Determining, quantifying, and qualifying this risk has implications for researchers, supply chain stakeholders, governments, and international policymakers in how they evaluate and target such issues through their activities.

Challenges for risk analysis include the sector’s scale, poor public data availability on medical product procurement, and associated risks or actual incidents of labour abuse [7]. However, existing guidance in ethical procurement suggests the risks of labour rights abuse are highest when manufacturing occurs in a country known to have weak legislation, policies, or a track record of protecting workers [8]. Thus, the country of origin (COO) of a product can serve as a suitable proxy for the risk of labour abuse and is a method already used by government and inter-governmental agencies to assess supply chain risk in other sectors. An analysis of 100,000 audits on the Sedex platform (a risk assessment tool built by a UK-based company using supplier environmental, social, and governance data) found that the COO was predictive of the risk of labour rights abuse [9].

Here, we quantify the risk of labour rights abuses in the manufacture of medical products supplied to a high-income country (Norway) based on product COO and compare this with COO data on medical products purchased in other high-income settings to assess the generalisability of our findings. We compare risk at the level of the whole dataset and for specific product categories. Specifically, we analysed four datasets on COO for medical products purchased for use in high-income countries: procurement data for South-Eastern Norway and Cambridge University Hospitals (England), global trade data from the UN Comtrade database, and registry data from the US Food and Drug Administration (FDA) database. We focused on high-income contexts due to the high level of consumption of medical goods in these countries and chose to focus on Norway given the quality of data available for this region. However, given the nature of globalised supply chains, our study is relevant to those in countries of all income levels who procure or supply medical products. We argue that if there is evidence of a prevalent risk of labour rights abuses across these multiple datasets, this indicates likely risk in the sector as a whole.

Parts of the work underlying this article were previously presented as a poster at the 2019 Public Health@Cambridge Network Showcase meeting on November 15, 2019, and as a conference abstract with an accompanying oral presentation at the 2021 Sustainable Healthcare Academic Research and Enterprise Conference on May 1, 2021. This article was also presented as an abstract with an accompanying oral presentation at the Brighton and Sussex Health Research Partnership conference on October 19, 2023.

## Materials and methods

Primary dataset: medical products purchased by the South-Eastern Norway Regional Health Authority

In the 2015-16 financial year (and only this year), suppliers to publicly funded hospitals in the South-Eastern region of Norway (around half of Norway’s population or >2.5 million people) were contractually required by the regional health authority (*Sykehusinnkjop*) to self-declare the production location of the main component of the products they supplied. This included products classified as 'medical consumables' or 'surgical products', excluding medicines, medical aids, laboratory products, and supplies through local contracts with individual hospitals. We will refer to this data as the ‘Norway dataset’.

These data were supplied to the senior author (MFB) on request (in accordance with Norwegian law that obligates the sharing of data related to public expenditure). This dataset was analysed using a stepwise approach. First, we assigned products to categories based on intended usage. Categories were not pre-specified, as there is no validated approach for assigning medical products to such categories, and the contents of the data were unknown prior to exploration. Aberrant or out-of-remit products, non-medical products (e.g., kitchenware, cleaning products, packaging), capital goods (e.g., sterilisation equipment), and medicines were excluded. We assigned the remaining product categories to ‘super-categories’ based upon similarities in material composition and physical design, on the assumption that such products would likely be manufactured in similar factories. For example, metal surgical instruments and metal laryngoscope blades are made in the same factories in Pakistan, and plastic kidney dishes and plastic specimen containers are made in the same factories in Mexico [10]. Multi-component products (e.g., procedure packs) were assigned a separate category. Efforts to reduce categorisation bias included cross-checking of excluded product categories and assignation to product super-categories by two authors (JA and MFB), with disagreements resolved by discussion.

For all products, we equated the self-declared location of production to the product COO. We determined a product to be at high-risk of labour rights abuse where the COO was one with known systematic violations of worker rights (rating 4) or no guarantee of rights (rating 5 or 5+) as defined by the International Trade Union Confederation (ITUC) Global Rights Index for 2016 [11]. This rating scale enables comparison of risk between datasets and utilises a variety of sources on real rights abuse instances alongside assessment of each country’s legal processes to inform contemporaneous risk ratings, minimising bias compared to supplier reporting [12,13]. We calculated the proportion of products at high-risk of labour abuse for all medical products and the proportion within each super-category.

The Norway data are, to our knowledge, the most comprehensive and granular data on the origin of medical products used within healthcare systems in high-income countries. To ascertain congruity and relevance to other contexts, we obtained data from three other high-income settings and cross-analysed the findings to compare the overall risk and the risk for specific product lines. We call these ‘comparative datasets’.

Comparative dataset on overall risk: hospital procurement data from Cambridge, England

We obtained procurement data for the 2018-19 financial year for Cambridge University Hospitals NHS Trust (a tertiary-level healthcare provider in England, providing core care to a local population of half a million and regional specialist services to six million) through finance (*Qlikview*, Qlik, Pennsylvania) and inventory (*Powergate*, GHX, Louisville) databases. We call this the ‘Cambridge dataset’. We extracted the top 100 items by spend under the categories 'medical or surgical equipment' and 'dressings', excluding capital purchases (which could skew data). For products in this dataset, we followed a standardised method of data collection. One author (JA) physically examined product packaging for statements in English on the country of manufacture (=COO), including statements using the terms 'made in', 'manufactured in', or 'assembled in' and any location identified through a manufacturer symbol and listed address. JA also searched supplier and manufacturer websites and performed Google searches using a systematic method to corroborate data on packaging. Where there was a discrepancy between the packaging and online sources, the COO on the packaging was used.

Mirroring our analysis for the primary Norwegian dataset, for the Cambridge dataset we matched COO to classification on the ITUC Global Rights Index but for 2019 [14] (the financial year of this analysis) to ascertain the overall proportion of goods classified as high-risk (ITUC rating 4, 5, or 5+). For multi-component products, we assigned the product to the highest ITUC rating for the COO of any of its components. Where we could obtain data on only some components, we recorded COO as 'partially available'.

Comparative datasets on risk for specific product lines: global trade data and US registry for medical products

We know of two public databases documenting the COO of products that contain data on some medical products: (1) the United Nations (UN) Statistics Division Commodity Trade database (UN Comtrade), built on import and export data supplied by member countries of the UN [15], and (2) the US FDA Medical Devices Product Registration database, which registers all producers or distributors of medical devices intended for sale in the USA (where registration is a legal requirement) [16]. From each database, we extracted information on medical products also present in the Norway dataset.

We searched the UN Comtrade database using the World Integrated Trade System (*WITS*, The World Bank, Washington, DC) online platform for exports in 2019. We identified medical products using World Customs Organisation HS 2017 nomenclature [17] and subtracted 're-exports' from 'gross exports' to calculate net exports. For countries with a net export, we assumed the product was manufactured in that country (=COO), and export volumes equated to the quantity manufactured and supplied to the global market. We matched the COO to the 2019 ITUC ratings [14] and calculated the proportion of products at high-risk of labour abuse (rating 4, 5, or 5+).

During October and November 2021, we conducted a search of records on the FDA database for all product categories that comprised >2.5% of contract value (spend) in the Norway dataset, aiming to identify all product codes related to that category. We extracted all 'contract manufacturers' or 'manufacturers' listed on the database under these product codes and equated the COO of the product to the manufacturer’s registered address. The FDA database does not include trade volumes. To estimate the proportion of a product coming from each country, we assumed every registered manufacturer of a particular product supplied equal volumes of that product. We matched the COO to 2021 ITUC ratings [18] and calculated the proportion of products at high-risk of labour abuse (rating 4, 5, or 5+).

For both comparator datasets, product codes were identified by one author (JA) with a review by a second (MFB). We compared the proportion of each product category or super-category in the Norway dataset to the equivalent product categories in other datasets.

## Results

Primary dataset

There were 23,972 products in the Norway dataset, 22,739 after excluding out-of-remit products (see the Appendices section for a list of out-of-remit products). COO was declared by the supplier for 55.4% of these by value (Table 1).

**Table 1:** COO of medical products supplied to the South-Eastern Norway Regional Health Authority in 2015-16 ranked by order of value of contracts (Euros, €) and proportion of the total value of all contracts The 2016 ITUC rating is also shown [11], and cells representing ITUC ratings of 4, 5, or 5+ are equated to a high-risk of labour abuse. A dash in the ITUC column represents there is no ITUC rating for that country or that the COO is unknown. Countries supplying <0.5% of the total value of contracts are not shown COO: country of origin, ITUC: International Trade Union Confederation

COO	Value of contract (€)	Proportion of total value (%)	ITUC rating (2016)
Not available	55,879,804	44.61	-
USA	21,802,238	17.41	4
Germany	5,167,919	4.13	1
France	4,642,393	3.71	2
Costa Rica	3,664,715	2.93	2
Malaysia	3,465,949	2.77	4
China	3,254,196	2.60	5
Ireland	2,968,309	2.37	1
Czech Republic	2,351,823	1.88	2
UK	2,149,468	1.72	3
Mexico	2,130,759	1.70	5
Japan	1,825,733	1.46	2
Switzerland	1,738,484	1.39	2
Canada	1,338,649	1.07	2
Hungary	1,323,035	1.06	3
Dominican Republic	1,204,289	0.96	2
Portugal	1,160,267	0.93	2
Singapore	1,028,794	0.82	2
Poland	1,025,388	0.82	3
Spain	969,137	0.77	3
Finland	942,033	0.75	1
Thailand	936,572	0.75	4
Sweden	909,776	0.73	1
Austria	703,751	0.56	1
Cambodia	423,026	0.34	5
Philippines	346,912	0.28	5
Slovakia	329,545	0.26	-
Denmark	275,063	0.22	1
India	263,448	0.21	5
Italy	238,267	0.19	1
Puerto Rico	187,891	0.15	4
Belgium	163,716	0.13	1
Netherlands	73,612	0.06	1
Swaziland	67,815	0.05	4
South-Korea	57,895	0.05	5
Total	125,249,863.57	100	

The most common COOs were the USA (17.4% of contracts by value), Germany (4.1%), France (3.7%), Costa Rica (2.9%), Malaysia (2.8%), and China (2.6%). All other countries had contracts worth <2.5% of the total. By continent, 24.2% of COOs were in North America, 21.7% in Europe, 9.3% in Asia, and 0.05% in Africa. Where COO was declared, the total proportion of purchases at high-risk of labour abuse was 49.1% (€34,028,154/€69,370,059 of purchasing), comprising 42.6% from countries rated ITUC 4 and 6.5% rated ITUC 5 (none rated ITUC 5+).

Products from the Norway dataset were classified into 294 categories and 17 super-categories (see the Appendices section for the value of contracts for product super-categories and the product categories they contain from the analysis of the Norway dataset). Table 2 lists the COO for products in each super-category and the proportion by value of the contract with a high-risk of labour abuse. While total purchase value and missing data varied, where COO was present, certain super-categories showed higher proportional risk (e.g., gloves (82.3%) and electrical peripherals (78.0%)), while others showed lower risk (e.g., procedure packs (6.7%), fluid-based (32.3%), and non-sheet plastic products (33.2%)). Within super-categories, levels of risk and missing data also varied; for example, super-category ‘textiles’ includes ‘operating drapes’ (70.5% by value high-risk, 21.5% missing), ‘surgical gowns’ (91.7% high-risk, 8.3% missing), and ‘uniform’ (3.8% high-risk, 6.7% missing).

**Table 2:** COO of medical products supplied to the South-Eastern Norway Regional Health Authority in 2015-16 grouped by product ‘super-categories’ and ranked by order of value of contracts (value in Euros, €) Countries supplying <2.5% of the value of contracts for that ‘super-category’ are not shown. COO is mapped to the 2016 ITUC country rating for labour rights risk [11], with an ITUC rating of 4, 5, or 5+ equated to a high-risk of labour abuse COO: country of origin, ITUC: International Trade Union Confederation

Product super-category	Percentage of total purchasing % (value in €)	COO	Value of contract (€)	Percentage of the total value of super-category (%)	ITUC rating (2016)	Percentage of purchasing where COO not declared % (value in €)	Percentage of purchasing where COO is declared with a high-risk of labour abuse % (value in €)
Cannulae, catheters, and balloons	12.2 (15,296,728)	USA	2,350,155	15.4	4	52.8 (8,071,637)	41.0 (2,962,727)
		Singapore	1,026,721	6.7	3		
		Germany	791,104	5.2	1		
		Ireland	711,654	4.7	2		
		Japan	617,749	4.0	2		
		Hungary	388,722	2.5	3		
Cement and scaffolds	0.9 (1,116,235)	France	122,501	11.0	1	86.9 (969,748)	2.8 (4,066)
Electrical devices	9.4 (11,824,629)	USA	2,316,909	19.6	4	51.0 (6,029,446)	47.3 (2,741,339)
		Costa Rica	2,270,162	19.2	2		
		Germany	783,681	6.6	1		
		Mexico	424,429	3.6	4		
Electrical peripherals and attachments	7.5 (9,419,939)	USA	3,212,528	34.1	4	43.3 (4,082,178)	78.0 (4,163,321)
		Poland	868,808	9.2	4		
		Germany	764,770	8.1	1		
Gloves	3.2 (3,962,190)	Malaysia	2,816,014	71.1	4	6.5 (257,844)	82.3 (3,046,988)
		Austria	657,358	16.6	1		
		Thailand	228,567	5.8	4		
Liquids, sprays, and gels	0.9 (1,078,793)	Portugal	217,327	20.1	2	28.8 (310,488)	32.3 (247,953)
		USA	164,559	15.3	4		
		Denmark	148,920	13.8	1		
		Puerto Rico	83,157	7.7	4		
		UK	59,068	5.5	3		
		Switzerland	50,874	4.7	2		
		Germany	40,938	3.8	1		
Metal implants	18.9 (23,668,647)	USA	6,882,686	29.1	4	49.1 (11,612,682)	58.1 (6,974,394)
		France	2,541,637	10.7	1		
		Ireland	862,379	3.6	2		
		Switzerland	590,019	2.5	2		
		Germany	578,366	2.4	1		
Metal instruments	2.5 (3,088,575)	USA	470,645	15.2	4	56.3 (1,737,362)	57.7 (780,391)
		Japan	309,658	10.0	2		
		Germany	160,941	5.2	1		
		Malaysia	155,074	5.0	4		
		Poland	106,580	3.5	4		
Multi-material products and miscellaneous	8.5 (10,584,943)	USA	2,390,187	22.6	4	47.4 (5,012,078)	49.7 (2,767,801)
		Dominican Republic	101,6301	9.6	2		
		Spain	859,242	8.1	3		
		Japan	296,900	2.8	2		
Needles	1.0 (1,241,097)	USA	243,316	19.6	4	66.0 (819,136)	71.6 (302,312)
		Japan	83,904	6.8	2		
		Mexico	58,995	4.8	4		
Non-metallic implants	4.0 (4,969,816)	France	692,041	13.9	1	60.5 (3,008,528)	37.9 (742,554)
		USA	690,079	13.9	4		
		Switzerland	248,949	5.0	2		
		Germany	165,014	3.3	1		
Non-sheet plastic	8.6 (10,744,866)	USA	1,115,132	10.4	4	40.0 (4,294,371)	33.2 (2,142,338)
		France	825,457	7.7	1		
		Germany	745,289	6.9	1		
		Finland	533,279	5.0	1		
		Switzerland	481,233	4.5	2		
		Portugal	454,265	4.2	2		
		Canada	438,468	4.1	2		
		Malaysia	347,865	3.2	4		
		The Philippines	346,912	3.2	5		
Plastic sheets, films, and bags	1.7 (2,164,665)	Mexico	520,853	24.1	4	29.3 (634,795)	53.2 (814,336)
		UK	244,660	11.3	3		
		Germany	182,740	8.4	1		
		USA	168,664	7.8	4		
		Slovakia	148,964	6.9	1		
		Dominican Republic	89,070	4.1	2		
		China	76,464	3.5	5		
Procedure packs and multi-component products	7.0 (8,821,407)	Czech Republic	2,105,346	23.9	2	42.2 (3,720,874)	6.7 (339,481)
		Ireland	840,147	9.5	2		
		Hungary	696,633	7.9	3		
		Portugal	465,183	5.3	2		
		France	264,940	3.0	1		
		Sweden	217,862	2.5	1		
Textiles	5.5 (6,842,401)	China	2,439,147	35.6	5	20.2 (1,383,064)	59.1 (3,225,590)
		Canada	807,812	11.8	2		
		Sweden	536,800	7.8	1		
		Cambodia	423,026	6.2	5		
		Mexico	267,741	3.9	4		
		Germany	225,584	3.3	1		
		UK	192,394	2.8	3		
		Switzerland	168,985	2.5	2		
Wires and stents	5.4 (6,801,498)	USA	868,533	12.8	4	46.7 (3,179,236)	44.6 (1,614,037)
		Costa Rica	806,685	11.9	2		
		Thailand	539,799	7.9	4		
		Ireland	488,399	7.2	2		
		Japan	295,671	4.3	2		
		Germany	286,097	4.2	1		
Wound dressings	2.9 (3,623,426)	UK	702,272	19.4	3	20.9 (756,331)	39.4 (1,128,733)
		USA	638,456	17.6	4		
		China	467,321	12.9	5		
		Finland	359,788	9.9	1		
		Switzerland	180,143	5.0	2		
		Germany	167,745	4.6	1		
		Costa Rica	156,818	4.3	2		

Comparative datasets

In the Cambridge dataset, four items (of 100) were excluded as capital goods. Packaging was available for 83/96 items and supplier and manufacturer websites for 95/96 items (one website was under maintenance). Seventy items (70/96; 73.0%) had information available on COO, including six multi-component products with partially available information (see the Appendices section for the item categories and associated annual spend for products included in the Cambridge dataset). For 10 products, online data were incomplete or did not match product packaging. Of the 70 products for which the COO was identified (Table 3), 52.7% of annual spend came from COOs Mexico, the USA, or China. Thirty-nine (39/70; 55.7%) products had a COO of high-risk of labour rights abuse (ITUC rated 4 = 17 products; 5 = 22 products), equating to 59.9% of products by value (ITUC rated 4 = 26.0%; ITUC rated 5 = 33.8%) where there was a COO available.

**Table 3:** COO of top 100 medical consumables (with four items excluded as per the main text description) by spend in the financial year April 2018-April 2019 supplied to Cambridge University Hospitals NHS Trust by annual spend (termed value of contracts, value in British Pounds, £) and ITUC rating In this dataset, for products of ‘multiple’ COOs, the highest ITUC-rated country is used to represent the product. COO is mapped to the 2019 ITUC country rating for labour rights risk [14], with ITUC rating 4, 5, or 5+ equated with a high-risk of labour rights abuse. Note: Malta and the European Community are not included in ITUC 2019 ratings, so the ITUC rating is listed as 'not recorded' COO: country of origin, ITUC: International Trade Union Confederation

COO	Number of products with COO	Summed value of contracts (£)	Proportion of total value (%)	ITUC rating (2019)
Unknown	26	3,713,620	27.0	-
Mexico	14	2,179,449	15.9	5
USA	12	1,981,895	14.4	4
China	7	1,126,414	8.2	5
Switzerland	6	734,879	5.4	2
Ireland	3	546,343	4.0	1
Malta	1	506,225	3.7	Not recorded
Malaysia	4	414,604	3.0	4
Costa Rica	2	323,040	2.4	2
Germany	3	272,188	2.0	1
Dominican Republic	2	247,268	1.8	2
Bosnia and Herzegovina	1	212,132	1.5	4
Israel	1	211,000	1.5	2
France	1	197,496	1.4	2
Estonia	2	182,710	1.3	2
UK	2	172,159	1.3	3
Spain	2	165,044	1.2	2
European Community	2	158,842	1.2	Not recorded
India	1	92,964	0.7	5
Australia	1	85,260	0.6	3
Singapore	1	79,200	0.6	2
Canada	1	72,964	0.5	2
Japan	1	68,536	0.5	2
Grand total	96	13,744,239	100	

Eight medical product codes were identified in the UN Comtrade database with equivalent products in the Norway dataset (see the Appendices section for the UN Comtrade product categories and codes). The COO for these products compared to the nearest equivalent product in the Norway dataset is summarised in Table 4.

**Table 4:** Comparison of COO for equivalent product categories/super-categories in the 2015-16 Norway and 2019 UN Comtrade datasets COO is compared for equivalent product categories/super-categories in the 2015-16 Norway dataset and the 2019 UN Comtrade database [15]. The first six columns relate to the Norway dataset: the name of the product category or super-category is shown in column 1 (with category/super-category in brackets indicating which of these is true for this group), columns 2-5 list the COO by contract value (in Euros, €) and the proportion of products from that COO alongside its ITUC rating, column 6 describes the overall proportion by value of that group from high-risk COO. The last six columns (columns 7-12) relate to data drawn from the UN Comtrade database, with column 7 listing the HS 2017 product code [17] used to search the database (shortened names are used in this table, see the Appendices section for the full category code name used in HS 2017 nomenclature). The value of net exports in column 9 is reported in US Dollars (USD), where a value of 1 in the table equates to 1000 USD. For both datasets, countries supplying <2.5% of the value of contract/net exports are not shown, percentage by value with a high-risk of labour abuse includes only products with a recorded COO (i.e., missing is excluded), and high-risk COO is defined as matching ITUC rating 4, 5, or 5+. COO is mapped to the 2016 (Norway data) and 2019 (UN Comtrade data) ITUC country rating for labour rights risk [11,14]. Note: the European Union is not included in the 2019 ratings and the value of contracts/exports values are listed in their respective reported currencies rather than being converted to a common denominator COO: country of origin, ITUC: International Trade Union Confederation

Norway data product (category/super-category)	Norway data: COO	Norway data: contract value per COO (€)	Norway data: percentage of total contract value per COO (%)	Norway data: ITUC rating (2016) of COO	Norway data: Percentage by value with a high-risk of labour abuse (COO ITUC 4, 5, or 5+) (%)	UN Comtrade product (HS 2017 code)	UN Comtrade data: COO	UN Comtrade data: net exports value per COO (1,000 USD)	UN Comtrade data: percentage of total net exports per COO (%)	UN Comtrade data: ITUC rating (2019) per COO	UN Comtrade data: Percentage by value with a high-risk of labour abuse (COO ITUC 4, 5, or 5+) (%)
Surgical gloves (category)	Malaysia	1,128,140	50.7	4	60.0	Surgical rubber gloves (401511)	Malaysia	377,704	25.2	4	66.4
	Austria	657,358	29.5	1			China	280,536	18.7	5	
	Not available	233,513	10.5	-			Thailand	271,886	18.1	4	
	Thailand	207,090	9.3	4			Germany	152,902	10.7	1	
							Belgium	85,834	5.7	2	
							European Union	60,487	4.0	-	
							Austria	53,973	3.6	1	
							Netherlands	49,074	3.3	1	
							India	39,261	2.6	5	
Cannulae, catheters, and balloons (super-category)	Not available	8,071,637	52.8	-	41.0	Catheters, cannulae, and the like (901839)	European Union	6,583,973	19.0	-	25.7
	USA	2,350,155	15.4	4			USA (and US territories)	5,195,227	5.0	4	
	Singapore	1,026,721	6.7	2			Netherlands	4,909,036	14.1	1	
	Germany	791,104	5.2	1			Ireland	4,212,700	12.1	1	
	Ireland	711,654	4.7	1			Germany	2,466,240	7.1	1	
	Japan	617,749	4.0	2			China	1,481,413	4.3	5	
	Hungary	388,722	2.5	3			Costa Rica	1,479,283	4.3	2	
							Belgium	1,330,903	3.8	2	
							Japan	1,066,423	3.0	2	
Syringes (category)	USA	742,500	28.7	4	57.1	Syringes, with or without needles (901831)	China	829,242	13.9	5	32.7
	Not available	682,668	26.3	-			USA (and US territories)	785,444	13.2	4	
	Canada	438,468	16.9	2			European Union	775,804	13.0	-	
	Philippines	346,912	13.4	5			France and Monaco	575,173	9.7	2	
	Switzerland	245,065	9.5	2			Germany	511,759	8.6	1	
	Belgium	80,596	3.1	1			Switzerland	350,883	5.9	2	
							Netherlands	288,602	4.9	1	
							Belgium	278,888	4.7	2	
							Italy	228,845	3.8	2	
Needles (super-category)	Not available	819,136	66.0	-	76.9	Tubular metal needles and needles for sutures (901832)	European Union	495,018	15.4	-	31.0
	USA	243,316	19.6	4			Belgium	331,923	10.4	2	
	Japan	83,904	6.8	2			USA (and US territories)	325,418	10.2	4	
	Mexico	58,995	4.8	5			China	270,886	8.5	5	
							Germany	216,340	6.6	1	
							Japan	209,084	6.5	2	
							Ireland	167,994	5.2	1	
							Denmark	114,561	3.6	1	
							Thailand	111,168	3.5	4	
							Singapore	98,877	3.4	2	
							Republic of Korea	105,562	3.5	5	
							Switzerland	98,877	3.1	2	
Adhesive dressings (category)	China	379,781	22.0	5	49.4	Dressings, adhesive, and other articles having an adhesive layer (300510)	European Union	777,116	16.6	-	25.7
	Not available	370,741	21.5	-			China	518,065	11.1	5	
	USA	288,679	16.7	4			Germany	480,313	10.3	1	
	Finland	180,570	10.5	1			UK	473,461	10.1	3	
	Germany	165,897	9.6	1			USA (and US territories)	379,833	8.1	4	
	Switzerland	155,838	9.0	2			Japan	258,519	5.5	2	
	UK	152,990	8.9	3			Belgium	201,514	4.3	2	
							France and Monaco	180,463	3.9	2	
							Netherlands	171,235	3.7	1	
							Hungary	169,465	3.6	3	
							Finland	159,636	3.4	1	
Non-adhesive dressings (category)	Not available	130,324	31.9	-	26.2	Wadding, gauze, bandages, and similar articles (300590)	China	1,040,541	21.7	5	31.5
	UK	74,544	18.2	3			European Union	782,434	16.3	-	
	China	58,651	14.3	5			UK	372,432	7.8	3	
	Norway	31,116	7.6	1			Germany	347,336	7.2	1	
	Canada	29,612	7.2	2			Netherlands	307,823	6.4	1	
	Finland	28,086	6.9	1			Belgium	303,799	6.3	2	
	Italy	19,052	4.7	1			Czech Republic	291,588	6.1	2	
	Hungary	18,461	4.5	3			USA (and US territories)	264,133	5.5	4	
							Ireland	145,708	3.0	1	
							France and Monaco	135,127	2.8	2	
Ultrasound and examination gel (category)	UK	33,336	45.7	3	0.0	Gel preparations designed to be used in human or veterinary medicine (300670)	European Union	63,135	17.8	-	38.0
	Not available	23,489	32.2	-			USA (and US territories)	59,606	16.8	4	
	Denmark	16,056	22.0	1			UK	28,026	7.9	3	
							Germany	23,719	6.6	1	
							China	22,135	6.3	5	
							Thailand	20,163	5.7	4	
							Ireland	18,777	5.3	1	
							Netherlands	18,532	5.2	1	
							Spain	12,994	3.7	2	
							Turkey	10,582	3.0	5	
							Italy	9,865	2.8	2	
							Austria	8,884	2.5	1	
Cardiac pacemakers and implantable defibrillators (category)	Not available	2,635,018	66.8	-	100	Pacemakers excluding parts and accessories (902150)	European Union	1,696,745	25.9	-	8.7
	USA	1,309,438	33.2	4			Ireland	1,098,954	16.7	1	
							Switzerland	827,188	12.6	2	
							Germany	813,276	12.4	1	
							Belgium	573,602	8.7	2	
							Malaysia	491,712	7.5	4	
							Netherlands	431,391	6.6	1	
							France and Monaco	310,200	4.7	2	

Codes for five medical products were identified in the FDA database (see the Appendices section for the FDA product category codes and descriptors). Table 5 summarises the COO for these products compared to the nearest equivalent in the Norway dataset.

**Table 5:** Comparison of COO for equivalent product categories between the Norway and FDA datasets COO is compared across equivalent product categories in the 2015-16 Norway dataset and the October/November 2021 FDA Medical Devices Database [16]. The first six columns relate to the Norway dataset: the name of the product category is shown in column 1 (no super-categories are included in this table); columns 2-5 list the COO and ITUC rating by contract value and the proportion of product by value (in Euros, €) from that COO; and column 6 describes the overall proportion by value of that product category from high-risk COO. The last six columns (columns 7-12) relate to data drawn from the October/November 2021 FDA Medical Devices Database: column 7 lists the category codes used to search the database for items equated to the Norway product category; columns 8-11 list the COO and ITUC rating by number of manufacturers listed and the proportion of manufacturers from that COO; and column 12 describes the overall proportion of manufacturers listed from high-risk COO. For both datasets, countries supplying <2.5% of the value of contract or number of total manufacturers listed are not shown, percentage by value with a high-risk of labour abuse includes only products with a recorded COO (i.e., missing is excluded) and high-risk COO is defined as matching ITUC rating 4, 5, or 5+. COO is mapped to the 2016 (Norway data) and 2021 (FDA data) ITUC country rating for labour rights risk [11,18]. See the Appendices section for the descriptors of FDA database category codes COO: country of origin, ITUC: International Trade Union Confederation, FDA: Food and Drug Administration

Norway product category	Norway data: COO	Norway data: contract value per COO (€)	Norway data: percentage of total contract value per COO (%)	Norway data: ITUC rating (2016) of COO	Norway data: percentage by value with a high-risk of labour abuse (COO ITUC 4, 5, or 5+) (%)	Equated FDA category codes with search results	FDA data: COO	FDA data: number of manufacturers listed per COO	FDA data: Percentage of all manufacturers per COO (%)	FDA data: ITUC rating (2021) of COO	FDA data: percentage of manufacturers with a high-risk of labour abuse (COO ITUC 4, 5, or 5+) (%)
Orthopaedic nails, plates and screws, including cannulas	Not available	6,905,008	83.6	-	53.7	DZL, GWO, GXN, HRS, HWC, JDS, NDF, NDH, NDJ, NQW	USA	3,644	59.7	4	66.7
	USA	723,143	8.8	4			Switzerland	1,021	16.7	2	
	Switzerland	454,446	5.5	2			Germany	528	8.7	1	
							France	250	4.1	2	
							China	205	3.4	5	
Electrosurgical products	USA	2,882,307	41.3	4	62.2	BWA, DWG, EKZ, FAR, FAS, FDI, FDJ, GEI, HGI, HQO, HQP, HQR, JOS, KGE, KNS, MUL, NEY, NLU, NTB, NUJ, NWI, OCL, ODR, ONQ, PUL, QAG, QEC	USA	1,198	52.0	4	63.3
	Not available	2,251,794	32.3	-			Germany	301	13.1	1	
	Poland	868,808	12.4	3			China	162	7.0	5	
	Germany	572,654	8.2	1			Mexico	135	5.9	3	
							Costa Rica	86	3.7	2	
							UK	60	2.6	3	
Hip prosthesis	France	2,107,199	37.8	2	26.5	JDD, JDG, JDI, JDL, KMC, KWA, KWB, KWL, KWY, KWZ, KXA, LPH, LWJ, LZO, LZY, MAY, MBL, MEH, MRA, NXT, OQG, OQH, OQI, OVO, PBI	USA	4,246	63.8	4	70.6
	USA	1,245,794	22.4	4			Switzerland	528	7.9	2	
	Ireland	861,543	15.5	1			Ireland	454	6.8	1	
	Not available	602,748	10.8	-			China	362	5.4	5	
	Germany	329,842	5.9	1			Germany	267	4.0	1	
	UK	290,366	5.2	3			UK	278	4.2	3	
							France	178	2.7	2	
Pacemakers and defibrillators	Not available	2,635,018	66.8	-	100	DRO, DTB, DXY, JOQ, KRG, LWP, LWS, MRM, NIK, NVN, NVY, OJX, OSR, OVJ, PNJ	USA	306	64.8	4	75.6
	USA	1,309,438	33.2	4			Malaysia	47	10.0	5	
							Switzerland	25	5.3	1	
							Germany	24	5.3	2	
							Ireland	18	3.8	1	
							Singapore	12	2.5	2	
							Mexico	12	2.5	3	
Clips and staples	USA	2,934,576	84.8	4	94.2	DSS, FZP, FZQ, GDT, GDW, HQW, JDR, MCH, MNU, NCA, NMJ, PKL	USA	422	56.8	4	67.0
	Not available	290,645	8.4	-			Mexico	105	14.1	3	
	Germany	104,993	3.0	1			China	56	7.5	5	
							Germany	44	5.9	1	
							Switzerland	31	4.2	2	
							France	21	2.8	2	

## Discussion

Risk of labour rights abuse in healthcare supply chains

This is the first study to comprehensively evaluate the risk of labour abuse in healthcare supply chains. The use of the Norway dataset alongside three comparative datasets on COO from different global contexts is a key strength of this study. The Norway data is a large sample containing granular information on product types, including volumes, value, and COO. The size and granularity of the Norway data increase its suitability in assessing risk for the region. The product types in the Norway data are likely similar for many high-income contexts, although this has not been formally assessed.

Data from the Norway and Cambridge datasets (Table 1, Table 3) indicate that for medical products with known COO, approximately half are manufactured in countries where there is a high-risk of labour rights abuse (COO rating of ITUC 4 or 5 at 49.1% of products by value in Norway and 59.9% in Cambridge). Across both datasets, the main countries contributing to this risk are the USA, Mexico, Malaysia, and China.

When this risk is broken down by product type (Table 2, Tables 4-5), the proportion of products manufactured in any country (and the proportion at high-risk of labour abuse) varied across datasets. For example, the only listed COO for cardiac pacemakers and implantable defibrillators in the Norway data is the USA (contract value €1,309,438), with the majority of COOs for this category missing. While the USA does make up the biggest number of manufacturers in the FDA data (Table 5), there are several other COOs listed here, and the UN Comtrade data (Table 4) does not include the USA as a COO. Variation in the proportion of each product type manufactured in any country likely relates to the supplier used (since suppliers use networks of manufacturers across different locations) and is also impacted by missing data. Moreover, individual supplier practices, including policies on preventing modern slavery and relations with manufacturers, will vary. For most products, therefore, it would be erroneous to generalise the level of risk from each category in the Norway dataset and make blanket statements on the level of risk: assessment should be related back to the supplier chosen for a product and their responsible business practices.

In contrast, our data show most medical gloves are manufactured in South and South-East Asia (Malaysia, Thailand, and China; Table 4), where issues of forced labour have long been documented. Therefore, this product category should be considered high-risk for the majority of suppliers. These issues were made evident during the COVID-19 pandemic when supply chains for personal protective equipment gained media and political interest, leading to the USA banning imports of gloves from several manufacturers in Malaysia, which in turn spurred improvements to working conditions [19]. While caution is needed in making comparisons between datasets to ascertain the specific levels of risk (as described above and in the following section), comparative datasets also suggest certain product categories, in addition to gloves, may be at higher (electrosurgical products, clips, and staples) or lower-risk (non-adhesive dressings and examination gel) of labour rights abuse, although even ‘lower-risk’ items contain significant contributions from countries with high ITUC ratings. We note that China is the top site of production of medical textiles in the Norway data (with contracts totaling €2.4 million), despite having been noted as a location for forced labour by Uyghur populations in picking cotton (although we recognise many medical textiles are made of plastic polymers) [20].

The only preceding study with an assessment of risk in healthcare supply chains is a recent policy paper from the NHS in England on the risk of modern slavery (including slavery, servitude, forced labour, and human trafficking) in its supply chain [21]. Predominantly informed by a supplier questionnaire, this assessment investigated whether companies reported supplying any of the five medical products previously highlighted (including by our group [10]) as at risk of modern slavery: face masks, gloves, gowns, surgical instruments, and uniforms. That report found 21% of suppliers to be at risk of modern slavery. In comparison, our study has looked at the proportion of products (not suppliers) at risk of labour abuse and includes the full range of medical products and all forms of labour abuse (beyond those that define modern slavery). Our approach enables the identification of risk in medical supply chains where products might not previously have been highlighted as high-risk of labour rights abuses and allows for variation in risk between suppliers supplying the same products. This is important given the dynamic nature of supply chains, where the supplier chosen is subject to constant change.

Limitations to assessment of risk

There are little or no data on actual working conditions at the site of manufacture for the products we have included, and so our findings are an estimation of labour abuse risk. While there are strong pieces of evidence for instances of labour rights abuses in medical product manufacture from case studies [1-3], we are not aware of previous studies that have analysed the COO of medical products and their associated risk of labour rights abuses. Countries rated by the ITUC as 4, 5, or 5+ show persistent or repeated failures to protect workers, and prior evidence suggests using COO is predictive of real-world labour rights issues [9]. However, there are caveats: where manufacturing is largely mechanised and automated, the risk of labour rights infringement is normally lower than where products are largely manufactured through unskilled labour [22]. Conversely, our methodology highlights the proportion of products from high-risk COOs (i.e., ITUC rating of 4 or more), but this does not exclude risk where the COO is one with a lower ITUC rating (e.g., ITUC rating of 3, which indicates known ‘regular violations of rights’).

Our study is a cross-sectional analysis where each dataset represents only a sample of activity within complex medical supply chains and has its own strengths and limitations. Differences in the construction and recording of these data between the four locations could limit comparison and generalisability. Although we compare the overall risk between the Norway and Cambridge datasets, the Cambridge dataset is a relatively small sample and derives from a specialist centre, which could have different procurement activities than other hospitals in England. There are also some unbalanced data contributions. For example, electrical devices, peripherals, and attachments constituted 50.5% of spend in the Cambridge dataset (see the Appendices section for the item categories and associated annual spend for products included with the Cambridge dataset), but only around 17% in the Norway dataset. We correlated the proportion of products at risk to expenditure, which could be unduly influenced by very high-cost items, such as cardiac pacemakers. The FDA database does not include data on quantity or value of trade; our assumption that manufacturers supply equal volumes is unlikely to reflect reality, and we recognise the limitations in comparing these proportions to the Norway data. Additionally, our assumption that export volumes in the UN Comtrade dataset equate to COO and quantity supplied to the global market may not always be true due to the complexities of global supply chains.

Our analysis relies on COO reporting by supply chain stakeholders, and our datasets contain incomplete or potentially inaccurate data. Importantly, we note the scale of the missing data. Suppliers to Norway did not declare the COO of their products for nearly half of the products by value, despite this being mandated. It is likely that in many instances, the supplier was not aware of where their product was manufactured or did not wish to declare it, circumstances often associated with poor labour practices [23]. It is possible, therefore, that the proportion of medical products at high-risk of labour abuse is even higher than estimated here.

Even where the COO was declared, this may represent a superficial or misleading statement. For example, the USA is a major site for the manufacture of medical products, supported by federal legislation that promotes the purchase of products wholly manufactured or substantially transformed in the USA. This policy does not, however, guarantee that products are actually manufactured in the USA; for instance, the medical suppliers Smith & Nephew and Medtronic have both received substantial fines for falsely stating medical products to be made in the USA when they were in fact manufactured in Malaysia or China [24,25]. We should also be concerned about multi-component products, where those components may come from different countries, but the country of final assembly is stated as the COO, masking the risk. In the Norway data, the Czech Republic was stated as the COO of nearly a quarter of procedure packs and multi-component products, yet other data in this paper suggest that the Czech Republic is not a manufacturer of the typical constituents of such packs (including gloves, gauze, cannulae, or catheters). We should also show caution towards electrical devices, peripherals, and attachments, whose manufacture is stated to be predominantly in the USA, Europe, and Central America. It is highly likely that some (or even the majority) of components for these products are manufactured elsewhere; for example, although USA companies are responsible for 49% of global sales of semiconductors, they only manufacture 12%, with most made in China and East Asia [26].

Finally, there is a risk of selection and misclassification biases in the methods to categorise products in the Norway dataset, to identify product COOs in the Cambridge dataset, and to identify equivalent codes in the comparator datasets. However, these should not significantly affect the assessment of overall risk, and the use of multiple datasets as comparators acts towards mitigating this.

Implications for research, policy, and practice

Our analysis provides strong evidence that many medical goods purchased in high-income countries are at high-risk of labour rights abuse in their manufacture. Around half of the products by value in the Norway data with known COO derived from countries at high-risk of labour rights abuse, constituting contracts of over €34 million in 12 months, and we found evidence of such risk in data from different global contexts. These contexts included a smaller procurement sample from an acute hospital in a high-income country where the overall level of risk was comparable to that in the Norway data and two large global trade datasets that demonstrated significant risk for specific product categories. Despite the limitations of our study, this suggests that the overall high-risk of labour rights abuses in the manufacture of medical products identified in the Norway data based on COO is likely representative of the sector. The EU recently published proposals for a new directive on mandatory human rights and environmental due diligence for companies established in the EU, recognising as high-risk sectors the textile, clothing, footwear, food, agriculture, forestry, fisheries, and extractive industries [27]. The data available to public buyers here establishes that medical goods are also a high-risk industry, implicating the need for coordinated, multi-disciplinary approaches involving researchers, policymakers, and supply chain stakeholders focusing on this sector.

Our study also highlights the potential utility of identifying high-risk products through COO as an initial approach to identifying risk, which could then be complemented by a rigorous investigation of potential high-risk areas [12]. However, in spite of our analysis being to date the most comprehensive assessment of the risk of labour rights abuses in medical supply chains, our attempts to identify high-risk products were limited by poor availability and transparency of COO data. The volumes of production by each COO and the exact level of risk for most products remain largely unknown. Further approaches to identifying high-risk products, including by ethical procurement practitioners, would require improvements to the availability and reliability of data on product COO. Thus, we believe that the proposed EU (and UK) diligence could be furthered by including the mandatory public declaration of product COO, which is already in place in countries such as Australia, Canada, South Korea, and the USA, but not the UK or EU [28]. Given the complexity of some products, in particular those with multiple components, the depth and granularity of transparency on COO will need to be sensitive to the particular product type and associated risk and also not simply focus on the first tier of a supply chain [12]. Transparency on COO will also help in quantifying and addressing environmental risks, given that typically 88% of the carbon footprint of single-use medical products is due to production [29].

Transparency in the COO should be seen as a first and necessary step in evaluating and tackling labour abuses in medical supply chains. What is also important is the governance in place to tackle such risks once identified, as well as the incentives and barriers to change through strategies of compliance or cooperation [30]. This would be complemented by further research into how supplier practices and supply chain stakeholders influence the risk of rights abuse, paying attention to the nuances specific to medical product supply chains. Our study does not include procurement data from middle- or low-income countries; future studies could assess if there are differences in the types and proportions of products purchased in these contexts and whether this impacts the level of risk taken on by the purchaser. Our analysis also did not include pharmaceuticals, but this is an important area for future research: major global sites of pharmaceutical production include China, India, and the USA, which are countries rated 4 or 5 on the latest (2023) ITUC Global Rights Index. Together, these approaches could enable a more comprehensive, validated assessment of risk at the level of specific medical products and specific supply chains, which would provide valuable information to supply chain practitioners and policymakers in all countries towards ending the practice of labour rights abuses in medical supply chains.

## Conclusions

There has been previous case-based evidence of labour rights issues in the manufacture of specific medical products, but our analysis (using multiple data sources) suggests high-risk across this entire sector. We add insight into the way that public procurers could undertake risk assessments of their medical supply chains and highlight limitations. Our findings demonstrate a critical and urgent need for greater transparency in global supply chains for medical products, which could be addressed through legislative or regulatory reform.

## Appendices

The following supplementary materials provide further supporting information and analysis to aid in the interpretation of the associated manuscript. As per the data availability statement, further original data and analysis are available upon reasonable request to the corresponding author. The appendices use the same nomenclature to refer to each dataset and reference as described in the main text. The methods used to obtain these data are described in the main manuscript.

**Table 6:** Excluded product categories from the Norway dataset Medical products supplied to the South-Eastern Norway Regional Health Authority in 2015-16, identified product categories that were excluded prior to analysis, and descriptors of those categories

Product category name	Category description
Alcohol gel	Alcohol gel for hand cleaning
Aluminium foil	Aluminium foil, generally used within a kitchen for food preparation/storage
Autoclave tape	Tape designed to be used to seal boxes to be put into an autoclave
Baby care products	Products including pacifiers, bottles, and nipple protectors designed for baby care. Excludes nappies
Baking paper	Baking paper for use in cooking
Barrier cream	Topical cream used to create a barrier between the skin and skin irritants
Blood bottles	Bottle for blood collection
Cardboard container	Cardboard container
Cleaning brush	Brushes for cleaning instruments and surfaces
Cleaning cloths	Cloths for cleaning instruments and surfaces
Clingfilm and clingfilm dispensers	Clingfilm and clingfilm dispensers
Coffee filter	Coffee filter
Cooking pans and tools	Cooking pans and tools
Crystalloid fluids	Crystalloid fluids for use in infusions
Cup holder	Holder for drinking cup
Cushion cover	Cover for cushions (furniture)
Cutlery	Cutlery
Disinfectant	Disinfectant liquids for cleaning surfaces and equipment
Draft barrier	Item for theatres. Excludes drafts from outside room
Equipment maintenance products other than specialist cleaning products, cleaning brushes, clothes, and oil	Equipment maintenance products other than specialist cleaning products, cleaning brushes, clothes, and oil
Floor mats and covers	Matts of varied materials for floor protection
Fortified drinks for patient consumption	Fortified drinks for patient consumption
Gloves dispenser	Dispenser for holding non-sterile gloves
Hairnets	Hairnets for use in food-handling areas
Hygiene bag dispenser	Dispenser for hygiene bags
Labels and label applicators	Labels and applicators for those labels, generally used to label boxes
Medications and products for injection	Medications and products for injection
Metal containers and instrument organisers	Metal containers and instrument organisers, e.g., as used for sterilisation
Microbiology products	Microbiology products, used during microbiological lab analysis of samples
Nail polish remover	Nail polish remover
Oil for equipment maintenance	Oil for equipment maintenance
Oral hygiene care (toothbrushes, picks, and others)	Patient products for oral hygiene care (toothbrushes, picks, and others)
Plastic packaging for sterilisation	Plastic packaging for sterilising equipment
Paper	Paper for general use
Paper towel	Paper towel
Parent code only	Products with parent code only, not a product in itself
Patient wristband	Wristbands for patient identification
Personal hygiene products (other than oral and toiletries)	Patient personal hygiene products (other than oral and toiletries)
Plastic bag	Plastic bag, unspecified use
Plastic containers	Plastic containers, unspecified use
Plastic cups	Plastic cups for drinking
Plates (for food)	Plates for food
Product for return	Unspecified product to be returned
Refrigerant	Refrigerant
Repair kit	Repair kit for equipment maintenance
Room lighting	Room lighting
Sack wire	Wire for closing bags
Sandwich bags	Paper/plastic bags designed to hold food contents
Shelving and racks	Shelving and racks
Shoe covers	Shoe covers, used to maintain area hygiene
Skin lotion and moisturiser	Skin lotion and moisturiser
Soap	Soap
Soap dispenser	Soap dispenser
Specialist equipment cleaning products	Specialist equipment cleaning products
Sterilisation indicator	Paper or other device that indicates when the sterilisation process is completed
Sterilisation machine component	Sterilisation machine component
Sterilisation paper	Paper used to wrap products for sterilisation
Table cover	Non-sterile table covers
Thermal cup	Cup for keeping contents at the desired temperature
Tissue dispenser	Tissue dispenser
Tissues and napkins	Tissues and napkins
Toiletries for patient care	Toiletries for patient care other than oral care products
Urine bottle stand	Urine bottle stand
Wet wipes	Wet wipes for non-medical purposes
Wall mount	Wall mount for device storage

**Table 7:** Value of contracts for product super-categories and the product categories they contain from the analysis of the Norway dataset Medical products supplied to the South-Eastern Norway Regional Health Authority in 2015-16 and the categories included in the final analysis, breakdown of assigned product categories contained within each super-category (super-category name and total contract value in bold), and the value of contracts per product category in Euros (€)

Super-categories and the product categories they contain	Contract value (€)
Cannulae, catheters, and balloons	15,296,728.50
Adaptors between tubes and syringes, plus end caps	822,041.49
Cardiac ablation catheters and related products	840,068.00
Cardiac catheters	2,994,374.48
Central arterial cannulae	54,167.00
Central venous cannulae	1,068,037.95
Endoscope sheaths and overtubes	84,216.10
Endoscope suction/biopsy channel	261,779.10
Endoscopic balloon catheter	613,860.92
Epidural catheters and other epidural products	458,060.77
ERCP catheter	65,596.30
Gastrostomy tubes and extension sets	1,479,875.75
Gel implant sizer	12,029.70
Haemostasis products (wound compression device)	558,464.70
Irrigation/suction/lavage catheter	538,921.08
Kit delivery catheters	6,458.00
Nasogastric, -duodenal, and - jejunal tubes	211,680.74
Occlusion catheters	230,622.13
Peripheral arterial cannulae	163,339.92
Peripheral venous cannulas	1,312,484.00
Rectal catheter	9,201.30
Spinal access ports	75.00
Spinal cannulae and lumbar puncture needles	58,746.78
Stent placement tools (endoscopy)	44,078.94
Surgical and non-surgical drains and drainage catheters	1,061,724.41
Uridom	239.56
Urinary catheters	518,643.23
Urinary dilatation catheters	29,265.70
Urinary occluder catheters	28,047.20
Uterine catheters	78,745.00
Vascular catheters	1,189,028.36
Veneports	186,448.00
Ventricular shunt and access ports	297,857.20
Vertebral body stents	18,549.70
Cement and scaffolds	1,116,235.17
Cement and related products/devices	890,398.17
Resorbable bone implants, bone matrix scaffolds	225,837.00
Electrical devices	11,824,629.67
Arthroscopic products	847,326.59
Cardiac pacemakers and implantable defibrillators	3,944,457.34
Computer for portable telemetry	166,200.23
Deep brain, spinal cord, and peripheral nerve stimulation	2,861,334.01
Electric device: irrigation, suction, and lavage	165,371.60
Electric drills	141,415.87
Electric saws	19,303.50
Electric thermometers	58,050.95
Endoscopes	84,200.00
Endoscopic capsule	291,110.90
Implantable cardiac monitor	684,055.14
Implantable drug pump	384,175.00
Infusion controller/pump	172,628.00
Invasive monitoring: pressure gauges and cable	824,774.41
Ophthalmoscope	8,393.00
Pen torches	19,769.60
Pulse oximeters	666,790.53
Ureteroscope	16,123.00
Ventricular assist device	469,150.00
Electrical peripherals and attachments	9,419,939.81
Battery packs	78,612.22
Bulbs for equipment	1,325.37
Defibrillator pads	111,556.61
ECG electrodes	426,138.22
ECG leads	75,970.59
Electric wires and cables	38,614.50
Electrosurgical grounding	5,054.40
Electrosurgical products	6,979,301.24
Endoscopic ultrasound and fine needle aspiration probes	3,144.78
Equipment and tools for use with cardiac leads and pacing devices	211,490.34
Fetal monitoring	4,713.80
Magnet for ICD deactivation	911.86
Neurosurgical electrodes	115,587.30
Ocular tonometry	268.20
Pacing leads	1,153,315.08
Pacing leads stylet	205.05
pH catheters	57,459.10
Pressure monitoring probes (manometry, intravascular, and intracranial)	119,793.15
Temperature probe for continuous monitoring	36,478.02
Gloves	3,962,190.74
Non-sterile examination gloves	1,736,087.81
Surgical gloves	2,226,102.94
Liquids, sprays, and gels	1,078,793.71
Gel for catheter insertion	25,955.00
Haemostasis products (liquid and gauze-based products)	562,638.05
Liquid wound closure preparations	131,266.47
Pastes, powders, and creams for stoma care	2,305.23
Pre-operative wash kit	238,179.29
Stoma bag removal spray	2.27
Ultrasound and examination gel	72,882.33
Wound cleaning products	45,565.06
Metal implants	23,668,647.81
Aneurysm clips	63,223.75
Clips and staples for surgical closure	3,458,589.13
Fixation and compression devices (orthopaedic)	277,479.11
Glenohumeral prosthesis	423,512.35
Hip prosthesis	5,567,753.07
Knee prosthesis	3,101,916.23
K-wire	192,063.10
Mechanical heart valve prosthesis	1,710,697.99
Metal orthopaedic implants other than prostheses	40,638.60
Nail end caps (orthopaedic)	50,927.05
Nuts, bolts, and washers	3,078.94
Orthopaedic nails, plates, and screws including cannulas	8,259,213.04
Other orthopaedic products	92,260.50
Wires and cables for orthopaedic procedures	427,294.95
Metal instruments	3,088,575.15
Biopsy curette	62.01
Biopsy forceps	352,654.10
Clamps	35,433.82
Laryngoscope	12,958.79
Manual drills and drill attachments	409,133.77
Manual saws and blades	860,653.16
Mixed orthopaedic tools	454,091.50
Obstetric forceps	2,594.30
Orthopaedic reamer	73,068.88
Orthopaedic templates	74.04
Skin graft carriers	11,721.80
Staple remover	701.42
Surgical retractors	76,946.59
Surgical tool organising	12,300.80
Surgical tools	775,643.81
Tuning fork	1,490.33
Vaginal specula	9,046.02
Multimaterial products and miscellaneous	10,584,943.16
Balloon inflation device	115,555.76
Biopsy gun/punch	161,448.72
Biopsy products, unspecified	2,987.53
Blood pressure cuffs	2,782.80
Blunt scissors	111,388.59
Cable and line organiser	157,627.41
Case for telemetry device	140,243.00
Catheter introducers and sheaths	833,809.34
Chin support	16,916.99
Clip applicator	391,218.49
CO2 insufflation needles	41,782.64
Cold/hot compress	79,565.97
Cutting block	260.71
Cytology brush	21,591.50
Depth gauge	49,297.50
Emergency blanket	535.87
Endoscopic ligation bands	44,624.88
Endoscopic tools	122,158.90
ENT stents	909.90
Filters for irrigation, suction, and lavage	29,463.62
Handles for irrigation/suction/lavage device	9,528.00
Identification loops for surgery	21,500.39
Infusion stand	9,733.70
Intraocular glide sheet	47.60
Laparoscopic accessories (excluding electrosurgical)	266,000.12
Ligamentous and tendon repair products	692,529.29
Marker pens	28,796.55
Medical tape	230,184.34
Nail brush	148,210.30
Ophthalmology eye spears	5,163.00
Ophthalmology lenses	18,576.90
Orthopaedic spacers	42,071.60
Patient warmers (blanket, self-warming)	38,318.00
Personal protective equipment: hoods	141,340.80
Plaster casts and related products	315,605.50
Pregnancy test	211.50
Repaired instruments	114,268.84
Safety pins	8,915.06
Sanding	28,004.40
Saw blade guard	843.90
Schirmer test paper	948.60
Screens for radiation protection	38,355.61
Screwdriver and screwdriver tips	12,266.51
Seal for irrigation, suction, and lavage	56.40
Shaving equipment	27,783.05
Smoke extraction device filter	50,154.00
Stapling machine	504,626.08
Sterile ruler	265.40
Stethoscopes	25,727.90
Suction system silencer	367.48
Supports and orthoses	234,116.27
Sutures	2,016,057.49
Tendon hammer	4,499.50
Tongue spatula	3,144.30
Tourniquet	53,915.30
Tracheostomy cleaning brush	7,420.97
Tracheostomy filter/vent	34,989.95
Tracheostomy pressure monitoring device	945.00
Trocars	1,843,273.77
Unknown	991,498.08
Ureteral access sheaths	186,115.00
Urinalysis kits	34,542.50
Urine bottle	10,224.49
Vomit bag	59,629.62
Needles	1,241,097.73
Biopsy needles	517,341.20
Endoscopy needles	221,012.50
Needles, unspecified	146,858.29
Suture needles	204,730.24
Transeptal needles	151,155.50
Non-metallic implants	4,969,816.21
Breast implants and tissue expanders	671,490.90
Endobronchial valves	55,796.40
ENT prosthesis	1,100.70
Nasal splints and septal buttons	1,957.40
Penile and testicular implants	104,199.60
Plastic orthopaedic implants other than prostheses	45,782.70
Septal occluders for ASD/VSD repair	154,370.00
Surgical mesh	1,197,600.89
Urethra slings	324,316.80
Vascular and cardiac repair grafts	2,413,200.82
Non-sheet plastic	10,744,866.57
Airways	625,903.04
Biopsy valves	294,640.90
Bite guard and tooth protectors	32,044.00
Breathing circuit	728,991.88
Breathing filters and exchangers	496,303.29
Catheter clamps	3,662.06
CO2 insufflation tubing	298,064.93
CPAP and BiPAP tubing and masks	471,982.77
Dosette box	6,830.22
Endoscope distal attachments	44,740.80
Fluid draining wick ophthalmology	42.00
Fluid warmer disposable cartridge	166,934.00
Guidewire delivery device	26,580.00
Hose for irrigation, suction, and lavage	557,175.85
Infusion burette	1,780.70
Infusion drip chamber and filters	678,705.04
Infusion tubing (without drip chamber, spikes, etc.)	946,118.61
Nasal cannulae	333,642.32
Nebulizer chambers and masks (not electrical devices)	127,334.90
Obstetric ventouse	466.00
Operation patient positioning	60,628.00
Otoscope disposables	23,874.74
Oxygen masks	192,975.92
Personal protective equipment: goggles and glasses	7,400.58
Plastic eye shield (post-ophthalmology surgery)	2,508.80
Rectoproctoscopes	64,202.40
Rigid collars and boards for trauma	37,714.31
Sharps bins	227,509.13
Specimen pots	37,398.61
Stoma bridge	17.20
Syringes	2,590,946.62
Tracheostomy tubes (inner and outer)	146,048.45
Tracheostomy valve	3,661.11
Uterine mobilisers	41,050.40
Valves for irrigation, suction, and lavage	27,057.56
Waste bins (other than sharps)	915,760.09
Water container for irrigation, suction, and lavage	524,169.36
Plastic sheets, films, and bags	2,164,665.83
Collection bag for stool	194,670.13
Corpse bag	9,405.90
Equipment covers	154,418.19
Infusion cuff	16,922.80
Laparoscopic wound protectors	13,841.50
Patient warmers (blanket, fillable)	422,696.86
Personal protective equipment: aprons	47,905.11
Personal protective equipment: face shields	4,822.38
Specimen retrieval bags	443,211.90
Stoma bags	83,577.65
Stoma rinsing sleeve	264.00
Ultrasound probe covers	198,101.94
Urinary catheter bags	380,527.47
Waste bags	194,300.00
Procedure packs and multi-component products	8,821,407.24
Catheter accessory kit	51,556.80
Cricothyroidectomy kit	3,122.86
Donation and cell-salvage kits	97,606.02
Irrigation, suction, and lavage kits	423,933.54
IV/SC administration set (including drip chamber, filters, spike, tubing)	1,690,497.36
PEG insertion kits	45,165.95
Procedure packs	5,932,723.83
Resuscitation kit	170.00
Robot-assisted surgery packs	48,642.00
Syringe kits	336,840.00
Tracheostomy insertion kit	5,5012.42
Tracheostomy suction kit	136,136.45
Textiles	6,842,401.28
Absorbent sheets for bed/chair protection	478,214.44
Alcohol wipes	25,033.38
Compression stockings	24,1652.16
Cotton wool and cotton buds	13,564.13
Fluid warmer strap	5,499.00
Menstrual and incontinence pads	734,143.27
Nappies	1,812.60
Neurosurgical patties	49,471.90
Operating drapes	1,598,803.67
Patient clothing	101,781.87
Patient transfer sheet	17,145.34
Patient warmers (other, textile)	236,990.64
Personal protective equipment: respirators	179,742.43
Personal protective equipment: surgical facemasks	225,270.76
Scrubs and uniform	361,179.10
Stoma belt	12,649.59
Surgical caps	209,063.52
Surgical gown	1,834,821.15
Surgical swabs	477,124.34
Tracheostomy neck strap	38,438.00
Wires and stents	6,801,498.92
Biliary and pancreatic stents	367,002.69
Breast tissue marker	13,913.00
Endovascular coils	384,950.90
Guidewires	2,143,142.71
Oesophageal and intestinal stents	258,222.00
Polypectomy wire	310,119.56
Renal stone basket	201,824.20
Sphincterotomes (for use in endoscopy)	539,595.91
Tracheobronchial stents	19,987.50
Ureteral stents	193,578.00
Vascular and cardiac stents	2,369,162.45
Wound dressings	3,623,426.08
Adhesive dressings	1,723,589.85
Epistaxis pack	18,747.20
Non-adhesive dressings	408,882.45
Specialised dressings	467,746.08
Stoma dressings	2,864.20
Stoma sealing rings	118.82
Vacuum dressings	1,001,477.48
Grand Total	125,249,863.57

**Table 8:** Item categories and associated annual spend for products included in the Cambridge dataset Breakdown by item category of the top 100 medical consumables (excluding four out of remit products as described in the main manuscript) by spend in the year April 2018-April 2019 supplied to Cambridge University Hospitals NHS Trust. The numbers of items in each category and the related annual spend in British Pounds (£) are provided. The item categories and super-categories are chosen based on the same categories used in the Norway dataset analysis, except for new categories of ‘other direct imaging equipment’, ‘organ transplant media’, and ‘wound care pack’ which have been assigned to appropriate super-categories. Super-categories and the total in that category are listed in bold, with item categories contributing to that category listed directly underneath

Super-category or product category	Number of items in the category	Annual spend of financial year 2018 (£)
Cannulae, catheters, and balloons	6	501,200.93
Cardiac catheters	1	68,536.17
Peripheral venous cannulae	1	112,690.48
pH catheters	2	158,644.00
Surgical and non-surgical drains and drainage catheters	1	88,504.32
Ventricular shunt and access ports	1	72,825.96
Electrical devices	28	4,355,974.40
Cardiac pacemakers and implantable defibrillators	11	1,932,464.50
Cochlear implant components	7	1,125,685.60
Deep brain, spinal cord, and peripheral nerve stimulation	4	543,618.00
Endoscopes	2	189,438.30
Other direct imaging equipment	1	211,000.00
Pulse oximeters	2	210,668.00
Ureteroscope	1	143,100.00
Electrical peripherals and attachments	14	2,587,145.51
ECG leads	1	90,045.00
Electrosurgical products	10	2,111,946.67
Fetal monitoring	1	115,057.84
Neurosurgical electrodes	1	72,600.00
Pacing leads	1	197,496.00
Gloves	6	573,951.63
Non-sterile examination gloves	4	412,416.68
Surgical gloves	2	161,534.96
Liquids, sprays, and gels	3	859,427.29
Haemostasis products (liquid and gauze-based products)	2	406,177.29
Organ transplant media	1	453,250.00
Metal implants	4	385,878.62
Clips and staples for surgical closure	4	385,878.62
Multi-material products and miscellaneous	11	1,345,357.75
Clip applicator	1	96,673.50
Laparoscopic accessories	5	585,399.03
Other equipment accessories	1	210,000.00
Stapling machine	2	275,887.23
Trocars	1	92,327.41
Urine bottle	1	85,070.58
Needles	1	77,550.00
Biopsy needles	1	77,550.00
Non-sheet plastic	11	1,296,972.14
Breathing circuit	3	257,014.00
Infusion tubing (without drip chamber, spikes, etc.)	3	537,675.45
Operation patient positioning	2	204,088.82
Syringes	3	298,193.87
Plastic sheets, films, and bags	1	71,318.40
Specimen retrieval bag	1	71,318.4
Procedure packs and multi-component products	7	1,246,935.16
IV/SC administration set (including drip chamber, filters, spike, tubing)	4	945,521
Neurosurgical shunt insertion kit	1	128,650
Procedure packs	1	92,964.16
Wound care pack	1	79,800
Textiles	2	254,157.75
Compression stockings	1	78,207.75
Operating drapes	1	175,950.00
Wires and stents	2	188,370.00
Sphincterotomes (for use in endoscopy)	1	120,120.00
Ureteral stents	1	68,250.00
Grand total	96	13,744,239.58

**Table 9:** UN Comtrade product categories and codes Medical product category names and code from HS 2017 [17] nomenclature that was explored and data obtained from UN Comtrade [15] via World Integrated Trade System (WITS, The World Bank, Washington, DC) online platform (as per method in associated manuscript). The table also describes the shorthand names used throughout the manuscript and supplementary information (which was generated by the authors) to refer to these categories

UN Comtrade category name	Shortened name used in these documents	Category code
Surgical rubber gloves	Surgical rubber gloves	401511
Medical, surgical instruments and appliances; catheters, cannulae, and the like	Catheters, cannulae, and the like	901839
Medical, surgical instruments and appliances; syringes, with or without needles (901831)	Syringes with or without needles	901831
Medical, surgical instruments and appliances; tubular metal needles and needles for sutures (code 901832)	Tubular metal needles and needles for sutures	901832
Dressings, adhesive; and other articles having an adhesive layer, packed for retail sale for medical, surgical, dental, or veterinary purposes (code 300510)	Dressings, adhesive; and other articles having an adhesive layer	300510
Wadding, gauze, bandages, and similar articles; (excluding adhesive dressings), impregnated or coated with pharmaceutical substances, packaged for retail sale (code 300590) (henceforth ‘non-adhesive dressings’)	Wadding, gauze, bandages, and similar articles	300590
Pharmaceutical goods; gel preparations designed to be used in human or veterinary medicine as a lubricant for parts of the body for surgical operations or physical examinations or as a coupling agent between the body and medical instruments	Gel preparations designed to be used in human or veterinary medicine	300670
Pacemakers; for stimulating heart muscles (excluding parts and accessories)	Pacemakers excluding parts and accessories	902150

**Table 10:** FDA product category codes and descriptors Medical product category codes from the FDA product code database [16] that were identified and data was collected as in Table 5 of the main manuscript. All these codes were those identified in searching the FDA product code database for equivalent product categories that comprised >2.5% of contract value for spend in the Norway dataset following the exclusion of out-of-remit products

FDA product code	FDA product description
MQZ	Prosthesis, nail
JDS	Nail, fixation, bone
NDH	Nail, fixation, bone, metallic
NDF	Plate, fixation, bone, non-spinal, metallic
HRS	Plate, fixation, bone
GXN	Plate, cranioplasty, preformed, non-alterable
GWO	Plate, cranioplasty, preformed, alterable
NQW	Orthosis, spine, plate, laminoplasty, metal
HWC	Screw, fixation, bone
NDJ	Screw, fixation, bone, non-spinal, metallic
DZL	Screw, fixation, intraosseous
FAR	Unit, electrosurgical
BWA	Unit, electrosurgical and coagulation, with accessories
EKZ	Unit, electrosurgical, and accessories, dental
KNS	Unit, electrosurgical, endoscopic (with or without accessories)
NLR	Unit, electrosurgical, endoscopic (with or without accessories), reprocessed
OEK	Water-induced thermotherapy system, benign prostatic hyperplasia
OCL	Surgical device, for cutting, coagulation, and/or ablation of tissue, including cardiac tissue
NEY	System, ablation, microwave and accessories
NTB	System, ablation, ultrasound and accessories
OEJ	Transurethral electrosurgical unit, benign prostatic hyperplasia
FDI	Snare, flexible
NLT	Snare, flexible, reprocessed
FDJ	Snare, rigid self-opening
PUL	Apparatus, cutting, radiofrequency, electrosurgical, AC-powered
NCR	Apparatus, cutting, radiofrequency, electrosurgical, battery-powered
NVJ	Applicator, transurethral, radio frequency, for stress urinary incontinence in women
FHZ	Desiccator, transurethral
JOS	Electrode, electrosurgical
FAS	Electrode, electrosurgical, active, urological
NLW	Electrode, electrosurgical, active, urological, reprocessed
ONQ	Electrosurgical coagulation for aesthetic
DWG	Electrosurgical device
NWI	Electrosurgical electrode kit
ODR	Electrosurgical patient return electrode
MUK	Electrosurgical radiofrequency system, stress urinary incontinence, female, transvaginal or laparoscopic, pelvic tissue
PDG	Electrosurgical vessel and/or tissue sealer. With built-in generator
GEI	Electrosurgical, cutting & coagulation & accessories
NUJ	Electrosurgical, cutting & coagulation accessories, laparoscopic & endoscopic, reprocessed
QAG	Endoscopic electrosurgical clip cutting system
KGE	Forceps, biopsy, electric
QEC	Forceps, biopsy, electric surgical hemostasis within tracheobronchial tree
NLU	Forceps, biopsy, electric, reprocessed
MUL	Generator, electrosurgical, coagulation, cancer
HGI	Electrocautery, gynecologic (and accessories)
HIM	Electrocautery, endoscopic and accessories
HQO	Unit, cautery, thermal, ac-powered
HQP	Unit, cautery, thermal, battery-powered
HQQ	Apparatus, cautery, radiofrequency, battery-powered
HQR	Apparatus, cautery, radiofrequency, ac-powered
NCR	Apparatus, cutting, radiofrequency, electrosurgical, battery-powered
PUL	Apparatus, cutting, radiofrequency, electrosurgical, AC-powered
OQG	Hip prosthesis, semi-constrained, cemented, metal//polymer, + additive, porous, uncemented
OQI	Hip, semi-constrained, cemented, metal/ceramic/polymer + additive, porous uncemented
OQH	Hip, semi-constrained, cemented, metal/polymer + additive, cemented
MAY	Prosthesis, hip, semi-constrained, metal/ceramic/polymer, cemented or non-porous cemented, osteophilic finish
KWZ	Prosthesis, hip, constrained, cemented or uncemented, metal/polymer
PBI	Prosthesis, hip, constrained, cemented or uncemented, metal/polymer, + additive
KXD	Prosthesis, hip, constrained, metal
JDG	Prosthesis, hip, femoral component, cemented, metal
KXA	Prosthesis, hip, femoral, resurfacing
KWB	Prosthesis, hip, hemi-, acetabular, cemented, metal
KWL	Prosthesis, hip, hemi-, femoral, metal
LZY	Prosthesis, hip, hemi-, femoral, metal ball
KWY	Prosthesis, hip, hemi-, femoral, metal/polymer, cemented or uncemented
JDH	Prosthesis, hip, hemi-, trunnion-bearing, femoral, metal/polyacetal
KXB	Prosthesis, hip, pelvifemoral resurfacing, metal/polymer
OCG	Prosthesis, hip, pelvifemoral resurfacing, metal/polymer, uncemented
JDL	Prosthesis, hip, semi-constrained (metal cemented acetabular component)
KWA	Prosthesis, hip, semi-constrained (metal uncemented acetabular component)
KMC	Prosthesis, hip, semi-constrained, composite/metal
OVO	Prosthesis, hip, semi-constrained, ceramic-on-metal articulation
LPF	Prosthesis, hip, semi-constrained, metal/ceramic/ceramic, cemented
MRA	Prosthesis, hip, semi-constrained, metal/ceramic/ceramic/metal, cemented or uncemented
LZO	Prosthesis, hip, semi-constrained, metal/ceramic/polymer, cemented or non-porous, uncemented
NXT	Prosthesis, hip, semi-constrained, metal/metal, resurfacing
JDI	Prosthesis, hip, semi-constrained, metal/polymer, cemented
LPH	Prosthesis, hip, semi-constrained, metal/polymer, porous uncemented
LWJ	Prosthesis, hip, semi-constrained, metal/polymer, uncemented
MEH	Prosthesis, hip, semi-constrained, uncemented, metal / polymer, non-porous, calcium phosphate
MBL	Prosthesis, hip, semi-constrained, uncemented, metal/polymer, porous
JDD	Prosthesis, upper femoral
NIK	Defibrillator, automatic implantable cardioverter, with cardiac resynchronization (CRT-D)
MRM	Defibrillator, implantable, dual-chamber
LWS	Implantable cardioverter defibrillator (non-CRT)
NVY	Permanent defibrillator electrodes
JOQ	Generator, pulse, pacemaker, external programmable (for electrophysiological studies only)
DXY	Implantable pacemaker pulse-generator
LWP	Implantable pulse generator, pacemaker (non-CRT)
PNJ	Leadless pacemaker
DRO	Pacemaker, cardiac, external transcutaneous (non-invasive)
OSR	Pacemaker/icd/crt non-implanted components
DTB	Permanent pacemaker electrode
KRG	Programmer, pacemaker
OVJ	Pulse generator, external pacemaker, dual chamber
HGB	Clip, tubal occlusion
DSS	Clip, vascular
PKL	Hemostatic metal clip for the GI tract
HQW	Clip, tantalum, ophthalmic
FZP	Clip, implantable
MCH	Clip, hemostatic
NCA	Clip, implantable, for coronary artery bypass graft (CABG)
NJC	Clip, vas deferens
NMJ	Clip, implantable, reprocessed
FZQ	Clip, removable (skin)
MNU	Staple, absorbable
JDR	Staple, fixation, bone
NDI	Staple, fixation, bone, metallic
GDW	Staple, implantable
NLL	Staple, implantable, reprocessed
GDT	Staple, removable (skin)
